# Metabolomic Analysis Reveals the Diversity of Defense Metabolites in Nine Cereal Crops

**DOI:** 10.3390/plants14040629

**Published:** 2025-02-19

**Authors:** Sishu Huang, Xindong Li, Kejin An, Congping Xu, Zhenhuan Liu, Guan Wang, Huanteng Hou, Ran Zhang, Yutong Wang, Honglun Yuan, Jie Luo

**Affiliations:** 1 National Key Laboratory for Tropical Crop Breeding, School of Breeding and Multiplication (Sanya Institute of Breeding and Multiplication), Hainan University, Sanya 572025, China; sishu.huang@hainanu.edu.cn (S.H.); lixindong@hainanu.edu.cn (X.L.); ansonakj@163.com (K.A.); zhenhuan.liu@hainanu.edu.cn (Z.L.); naturehht@foxmail.com (H.H.); zhangran@hainanu.edu.cn (R.Z.); wangyutong@hainanu.edu.cn (Y.W.); 2 School of Life Science and Technology, Wuhan Polytechnic University, Wuhan 430023, China; foreverxcp@126.com; 3 Yazhouwan National Laboratory, Sanya 572025, China; wangguan@yzwnationallab.cn

**Keywords:** cereal crops, defense metabolite, metabolomic, flavonoid, benzoxazinoid

## Abstract

Cereal crops are important staple foods, and their defense metabolites hold significant research importance. In this study, we employed LC-MS-based untargeted and widely-targeted metabolomics to profile the leaf metabolome of nine cereal species, including rice, wheat, maize, barley, sorghum, common oat, foxtail millet, broomcorn millet, and adlay. A total of 9869 features were detected, among them, 1131 were annotated, encompassing 18 classes such as flavonoids, lipids, and alkaloids. Results revealed that 531 metabolites were detected in all species, while each cereal crop possessed 4 to 12 unique metabolites. Focusing on defense metabolites, we identified eight benzoxazinoids uniquely present in maize, wheat, and adlay. Hierarchical clustering based on metabolite abundance divided all metabolites into nine clusters, and subsequent pathway enrichment analysis revealed that the stress-related flavonoid biosynthesis pathway was enriched in multiple clusters. Further analysis showed that four downstream compounds of HBOA (2-hydroxy-1,4-benzoxazin-3-one) in the benzoxazinoid biosynthesis pathway were enriched in maize. Wheat uniquely accumulated the 4′-methylated product of tricin, trimethoxytricetin, whereas adlay accumulated the tricin precursor tricetin in the flavonoid biosynthesis pathway. In summary, this study elucidates the metabolic diversity in defense metabolites among various cereal crops, providing valuable background information for the improvement of stress resistance in cereal crops.

## 1. Introduction

Cereal crops, including rice (*Oryza sativa* L.), wheat (*Triticum aestivum* L.), maize (*Zea mays* L.), barley (*Hordeum vulgare* L.), and sorghum (*Sorghum bicolor* L.), were domesticated as early as ten thousand years ago and represent a primary source of human calories [1,2]. In addition to providing essential food energy, certain cereals exhibit high nutritional value. For instance, adlay (*Coix lacryma-jobi* L.) contains a higher protein content compared to rice, wheat, and maize [3], while common oats (*Avena sativa* L.) are among the richest sources of not only proteins but also fats and vitamin B1 [4]. Due to their significant importance to humans, cereal crops have become the focus of numerous research efforts [5,6]. Among these, research on stress tolerance in cereal crops has emerged as one of the key focal areas [7,8].

Existing reports indicate that cereal crops exhibit diversity in terms of stress tolerance: for example, rice is a high-water-consuming plant and is highly sensitive to drought [9,10]. In contrast, crops such as foxtail millet (*Setaria italica* L.), broomcorn millet (*Panicum miliaceum* L.), and sorghum exhibit good drought tolerance, supporting their distribution in arid regions [6,10,11]. Additionally, crops have varying temperature requirements. Wheat, barley, and common oats being cool-season crops, are susceptible to high temperatures [4,12,13], while rice is more sensitive to cold stress compared to other cereal crops like wheat and barley [7]. Some cereal crops that grow in harsh environments, such as Tibetan hulless barley (*Hordeum vulgare* L. var. *nudum*), can resist high-intensity UV-B radiation and low atmospheric pressure [14]. Furthermore, plants are threatened by various pests and diseases, including the fungus *Magnaporthe oryzae* (anamorph *Pyricularia oryzae*) which causes rice blast disease [15], and rust fungi of the genus *Puccinia* which threaten wheat, barley, and common oat, leading to various rust diseases [16]. Clearly, different cereal crops possess distinct stress response mechanisms.

In cereal crops, the diversity of defense metabolites may influence the diversity of their stress responses. Some defense metabolites that share core structures are widely present across various plant species, although their diversity largely arises from chemical modifications of core structures [17,18]. For example, metabolites with diverse structures within the phenylpropanoid pathway are extensively involved in the biotic and abiotic stress defense processes of various plant species [19,20]. For instance, sakuranetin, a flavanone, is induced under phosphorus deficiency in rice and contributes to enhancing rice resistance to *Magnaporthe oryzae* under phosphorus-deficient conditions [21]. Additionally, the accumulation of several flavonoids can improve crop tolerance to UV-B radiation in rice [22]. Notably, the increasing content of flavonoids and anthocyanins enhances drought stress tolerance in Tibetan hulless barley [23]. Moreover, phenolamines, another type of phenylpropanoids, have been found in tomatoes to enhance drought tolerance [24]. CPH, a phenolamine in tobacco, has been found to play an important role in tobacco’s chemical defense against leafhoppers [25]. Beyond phenylpropanoids, other substances also widely participate in plant stress responses, including terpenoids [26,27,28,29], alkaloids [30,31], and branched-chain amino acids [32,33]. Similarly, several taxonomically restricted compound classes also have important roles in stress resistance. For example, glucosinolates in *Brassicaceae* plants are involved in defense against herbivores and pathogens [34]; steroidal glycoalkaloids (SGAs) in *Solanaceae* plants help protect against predators [35]; and isoflavones enhance leaf resistance to soybean (*Glycine max* L.) mosaic virus [20]. In some cereal crops, such as maize, wheat, and rye, a class of nitrogen-containing heterocyclic compounds called benzoxazinoids is present [36]. Benzoxazinoids act as allelochemicals, participating in early growth competition with other plants in maize, wheat, and rye, and they are actively involved in regulating plant resistance to pests and diseases [37]. Additionally, benzoxazinoids produced by cereal crops can be absorbed by neighboring plants and utilized to enhance their resistance against pests and pathogens [38]. In recent years, many defense metabolites in plants have been discovered, particularly within individual species, and the natural variation of these metabolites within species has led to differences in their defense capabilities [25,39]. However, analyses of the differences in defense metabolites among different cereal crops remain insufficient.

In this study, we employed metabolomics by LC-MS to analyze the leaf metabolomes of nine cereal crops, examining the similarities and differences in the metabolomes of cereal crops. We focused on analyzing the differences in defense substances such as benzoxazinoids and flavonoids among various cereal crops, thereby revealing potential differences in stress resistance mechanisms at the metabolic level. Our study provides a metabolic basis for understanding the resistance differences mediated by defense metabolites among cereal crops, thus offering valuable background information for the breeding and genetic improvement of stress resistance in cereal crops.

## 2. Results

### 2.1. Metabolomics Analysis of Nine Cereal Crops

**Figure 1:**
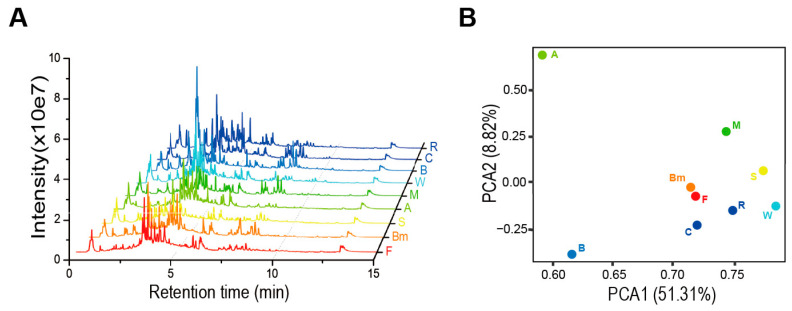
Overview of metabolic variation in nine cereal crops. (**A**) Total ion chromatography of metabolites in nine cereal crops based on untargeted metabolomics. (**B**) Principal component analysis (PCA) of all features detected in nine cereal crops. F, foxtail millet; Bm, broomcorn millet; S, sorghum; A, adlay; M, maize; W, wheat; B, barley; C, common oat; R, rice.

To comprehensively evaluate the metabolic differences among cereal crops, we conducted an untargeted metabolomic analysis using LC–TOF–MS on the leaves of nine cereal crops. To avoid bias toward specific metabolites introduced by the mass spectrometry scanning mode, we performed full scans of the pooled samples from three biological replicates for each species in both positive and negative modes separately. By comparing the total ion chromatogram (TIC), we observed significant differences among the various cereal crops, particularly in the signal-dense region between 2 and 4 min (Figure 1A).

Next, we performed peak detection and alignment on the untargeted data using MS-DIAL [40], resulting in a total of 9869 signals. Based on these signals, we conducted an unsupervised principal component analysis (PCA) on the nine cereal crops (Figure 1B). The results indicated that PC1 and PC2 explained 51.3% and 8.8% of the total variance, respectively. The PCA score plot revealed that adlay and barley were distinctly separated from the other crops along PC1, and adlay was also distinctly separated from all crops except barley along PC2, indicating significant metabolic differences between these two species and the others. In contrast, seven crops—rice, wheat, maize, sorghum, common oats, foxtail millet, and broomcorn millet—clustered closely together in the PCA score plot, particularly foxtail millet and broomcorn millet.

To address the limitations in sensitivity and quantitative accuracy inherent to untargeted metabolomics [41] and to obtain more precise quantitative results, we constructed an MS² spectral tag (MS2T) library based on the untargeted metabolomics data, and developed a widely targeted metabolomics method [42] to perform the quantitative analysis.

### 2.2. Metabolite Identification of Nine Cereal Crops

**Figure 2:**
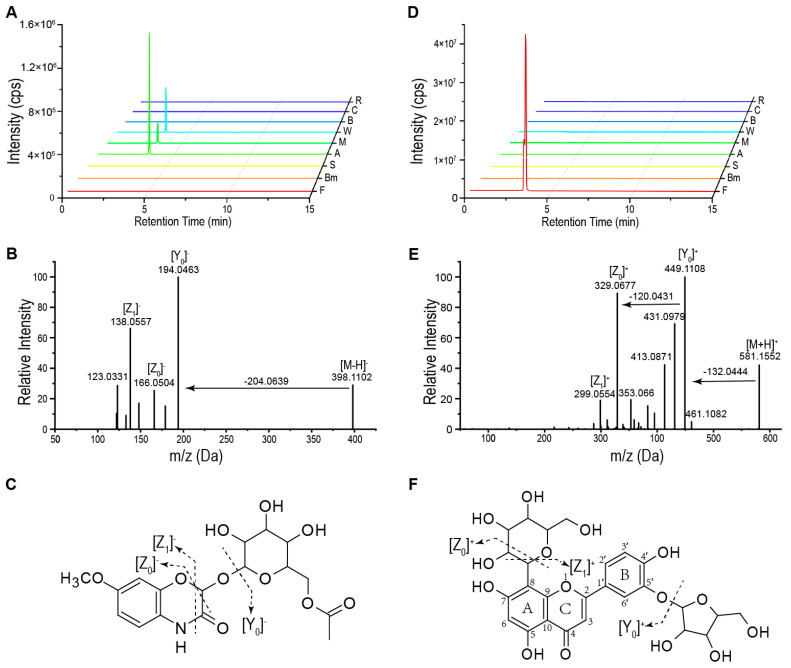
Annotation of CL1148 and CL0463 as HMBOA-acetyl-glucose and C-glucoside luteolin O-pentose, respectively. (**A**) Extracted ion chromatogram (EIC) of CL1148, a specific metabolite in maize, wheat, and adlay. F, foxtail millet; Bm, broomcorn millet; S, sorghum; A, adlay; M, maize; W, wheat; B, barley; C, common oat; R, rice; Sb, soybean; (**B**) MS/MS spectra of CL1148, which was identified as HMBOA-acetyl-glucose. (**C**) Putative fragmentation rules of HMBOA-acetyl-glucose. (**D**) EIC of CL0463, a specific metabolite in foxtail millet. F, foxtail millet; Bm, broomcorn millet; S, sorghum; A, adlay; M, maize; W, wheat; B, barley; C, common oat; R, rice; Sb, soybean; (**E**) MS/MS spectra of CL0463, which was identified as C-glucoside luteolin O-pentose. (**F**) Putative fragmentation rules of C-glucoside luteolin O-pentose.

We conducted a qualitative analysis based on the fragmentation patterns of the metabolites: benzoxazinoids are a class of nitrogen-containing heterocyclic compounds that typically exist in plants as glycosides [37]. For common benzoxazinoid aglycones, fragmentation of the deprotonated DIMBOA (2,4-dihydroxy-7-methoxy1,4-benzoxazin-3-one) typically yields fragments at *m*/*z* 166 and *m*/*z* 164, corresponding to neutral losses of CO_2_ (44 Da) and CH_2_O_2_ (46 Da). DIBOA (2,4-dihydroxy-1,4-benzoxazin-3-one) exhibits losses of CH_2_O_2_ (46 Da) to form a fragment at *m*/*z* 134. Typical fragments described for the [M − OH]^−^ ion of HBOA (2-hydroxy-1,4-benzoxazin-3-one) and HMBOA (2-hydroxy-7-methoxy-1,4-benzoxazin-3-one) are fragments corresponding to the loss of CO (28 Da), CH_2_O_2_ (46 Da), and 2CO (56 Da) [43]. For example, metabolite CL1148 (RT 3.39 min) is present in maize, wheat, and adlay (Figure 2A). CL1148 yielded a precursor ion [M − H]^−^ at *m/z* 398.1102. The tandem mass spectrum of CL1148 showed a characteristic fragment Y_0_^−^ ion at *m/z* 194.046, along with fragments of Z_0_^−^ ion at *m/z* 166.0504 and Z_1_^−^ ion at *m/z* 138.0557, which indicated the presence of an HMBOA skeleton (Figure 2B). The main neutral loss observed was 204 Da at *m/z* 194.0463, and we hypothesize that this loss corresponds to an acetyl-hexose (42 + 162 Da). Consequently, we identified CL1148 as HMBOA-acetyl-hexose (Figure 2C). Through fragmentation analysis of benzoxazinoids, we identified a total of eight benzoxazinoids.

Flavonoids are synthesized through the condensation of hydroxycinnamic acid (carbon atoms 2, 3, and 4 of the B and C rings) with a propylenol residue (A ring) and are primarily classified into classes such as flavones, flavonols, flavanones, flavanols, and anthocyanins based on variations in the C ring [44]. In mass spectrometry, the 1 and 3 positions of the flavonoid C ring are the primary cleavage sites. Additionally, hydroxyl substitutions frequently occur at the 5 and 7 positions of the A ring, resulting in [M + H]^+^ ions that typically produce a characteristic fragment at *m/z* 153. Certain flavonoids, due to the direct C-C bond linkage between the sugar moiety and the flavonoid aglycone at the A ring, undergo cleavage at the sugar substituent, generating characteristic ions such as [M + H − 150]^+^, [M + H − 120]^+^, and [M + H − 90]^+^. For example, metabolite CL0463 is a compound specifically present in foxtail millet (Figure 2D). CL0463 yielded a precursor ion [M + H]^+^ at *m/z* 581.1522. The Y_0_^+^ ion at *m/z* 449.1108 [M + H − 132]^+^ corresponds to the loss of a pentose. Additionally, the Z_0_^+^ ion at *m/z* 329.0677 and the Z_2_^+^ ion at *m/z* 299.0554 correspond to the neutral losses of 120 Da and 150 Da, respectively. Subsequently, a series of fragments of *m*/*z* 431.0979 and *m/z* 413.0871 corresponded to the loss of H_2_O. Therefore, we propose that this compound is a C-glucoside luteolin O-pentose (Figure 2F). As indicated by the untargeted data, a substantial number of flavonoids are present in cereal crops, and based on existing standards, we annotated a total of 311 flavonoids.

**Figure 3:**
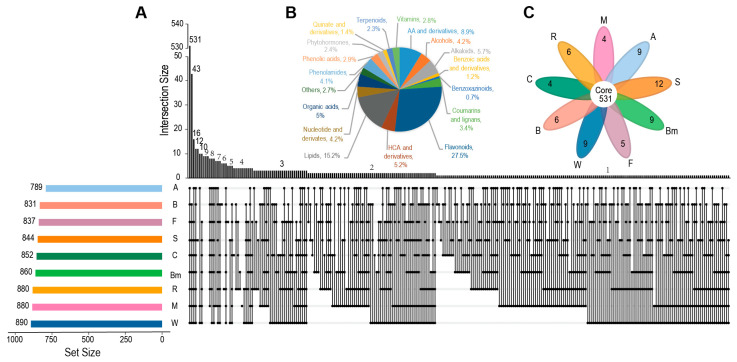
Classification and species distribution of 1131 annotated metabolites. (**A**) An upset plot of the number of annotated metabolites in nine cereal crops. (**B**) Classification of annotated metabolites. AA, amino acid; BA, Benzoic acid; HCA, hydroxycinnamic acid. (**C**) Venn diagram analysis of annotated metabolites. F, foxtail millet; Bm, broomcorn millet; S, sorghum; A, adlay; M, maize; W, wheat; B, barley; C, common oat; R, rice.

Using a similar approach and comparing to the standard, we annotated a total of 1131 metabolites by LC–MS/MS analysis, encompassing a wide range of metabolic classes in plants. In addition to the flavonoids and benzoxazinoids mentioned above, we also annotated 172 lipids, 101 amino acids, 64 alkaloids, 59 hydroxycinnamoyl derivatives, 57 organic acids and derivatives, 48 nucleotides and derivates, 48 alcohols and polyols, 46 phenolamides, 39 coumarins and lignans, 33 phenolic acids, 32 vitamins, 27 phytohormones, 26 terpenoids, 16 quinate and derivatives, 14 benzoic acids and derivatives, among 30 other compounds that did not fit into these 17 main classes (Figure 3B, Table S1).

### 2.3. Differentially Analysis of Metabolome Data

#### 2.3.1. Metabolite Composition Differences Among Nine Cereal Crops

To better understand the metabolic diversity of the nine cereal crops, we analyzed their metabolite compositions using LC-QTRAP-MS-based widely-targeted metabolomics method. Among the 1131 targeted metabolites, wheat had the highest number of detected metabolites (890), followed by rice and maize (880). The numbers of detected metabolites in the other cereal crops were as follows: 860 in broomcorn millet, 852 in common oat, 844 in sorghum, 837 in foxtail millet, 831 in barley, and 789 in alday (Figure 3A). A total of 530 metabolites were present in all nine species (shared metabolites), accounting for 46.5% of the total metabolites (Figure 3C). Among these, there were 315 primary metabolites (including lipids, amino acids, nucleotides, organic acids, and alcohols), representing 59.3% of the shared metabolites. And there were 216 shared secondary metabolites, representing 40.7%. We analyzed the proportion of each metabolite class relative to the total detected metabolites within that class among the shared metabolites to preliminarily assess the diversity of these metabolite classes across different species. Within the secondary metabolites, flavonoids accounted for the lowest proportion of the detected flavonoids at 12.5% (39 out of 311), followed by phenolamines at 15.2%. Other secondary metabolites ranged from 30.8% to 64.1%, while shared primary metabolites ranged from 54.2% to 84.3% of the detected metabolites (Table S2).

Additionally, we further analyzed the 600 metabolites that were missing in at least one cereal crop (non-shared metabolites). We first identified metabolites that appeared exclusively in a single cereal crop (species-specific metabolites). There were 12 metabolites detected exclusively in sorghum, and the fewest species-specific metabolites were found in maize and common oats, with 4 each. The number of species-specific metabolites detected in other species was as follows: 9 in wheat, 6 in barley, 5 in foxtail millet, 9 in broomcorn millet, and 9 in adlay (Figure 3C). Among the species-specific metabolites, flavonoids were the most abundant, with each species having between 1 and 7 specific flavonoids (Table S3). Additionally, we analyzed metabolites that were not detected in individual species (species-missing metabolites) to identify potential pathway deficiencies specific to those species. There were 43 species-missing metabolites in adlay, including 21 flavonoids and 1 to 4 metabolites from other classes. Subsequently, 16 species-missing metabolites were found in barley, 12 in foxtail millet, 8 each in maize, wheat, and rice, 7 in common oat, 6 in sorghum, and 3 in broomcorn millet (Figure 3A, Table S4).

**Figure 4:**
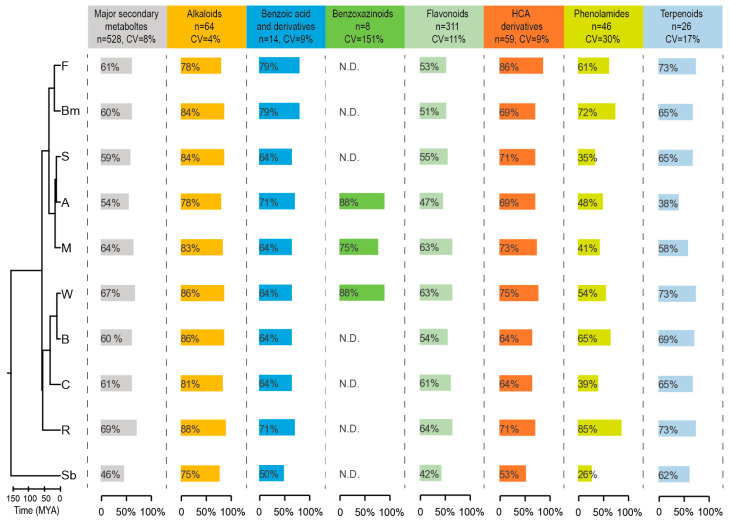
Treemap visualization of major secondary metabolite classes distribution. The lineage divergence time is adopted from the TimeTree database (http://www.timetree.org, accessed on 20 December 2024). F, foxtail millet; Bm, broomcorn millet; S, sorghum; A, adlay; M, maize; W, wheat; B, barley; C, common oat; R, rice; Sb, soybean; MYA, millions of years; N.D., non-detected.

To further analyze the variation of major defense metabolites among the nine cereal crops, we integrated the detection proportions of major secondary metabolite classes for each crop (the proportion of detected metabolites within each class relative to the total metabolites in that class) and mapped them onto the phylogenetic tree (Figure 4, Table S5). We calculated the coefficient of variation (CV) for the detection proportions of each metabolite class across the species to quantify the differences in the number of these metabolites among cereal crops. The analysis revealed that benzoxazinoids had the highest CV at 151% among the nine cereal crops, followed by phenolamines at 30%, terpenoids at 17%, and flavonoids at 11%. Further analysis showed that eight benzoxazinoids were uniquely present in maize, adlay, and wheat, with each species detecting at least six of these compounds and five being present in all three species (Figure 4). Additionally, phenolamines had lower detection proportions in sorghum, common oat, and maize (35%, 39%, and 41%, respectively), while they were detected at higher proportions in rice and adlay (85% and 72%, respectively). Terpenoids had lower detection proportions in adlay and maize (38% and 58%, respectively).

#### 2.3.2. Metabolite Abundance Differences Among Nine Cereal Crops

**Figure 5:**
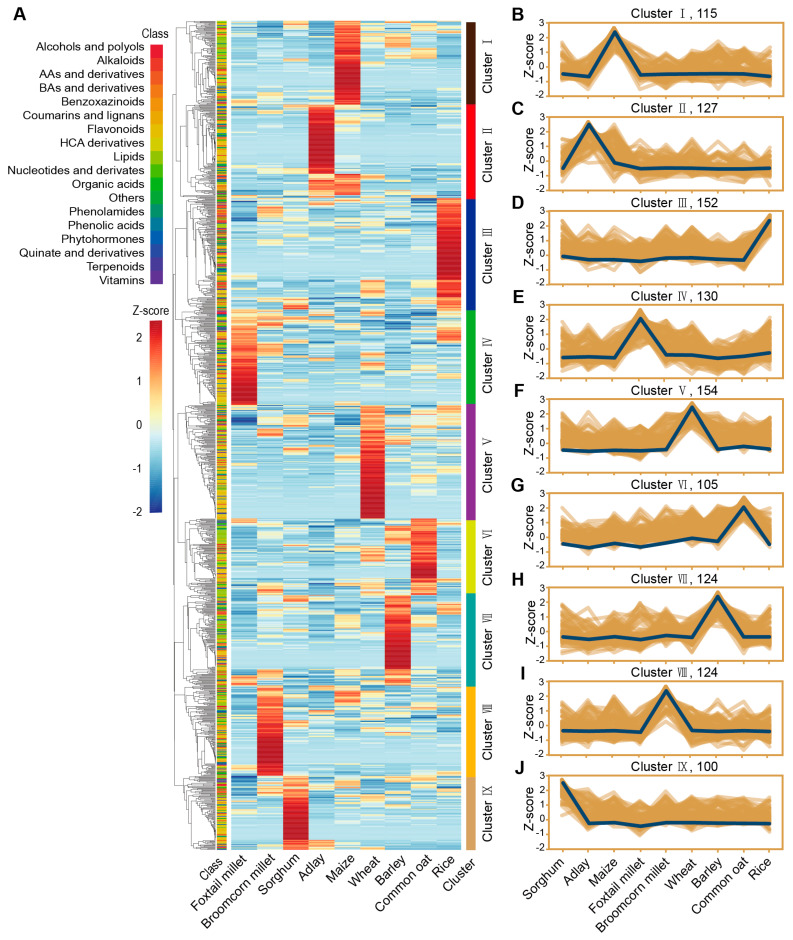
Hierarchical cluster analysis of 1131 annotated metabolites. (**A**) Hierarchically clustered heatmap of the 1131 annotated metabolites from nine cereal crops. Metabolic data were Z-score standardized (color scale from blue to red). AA, amino acid; BA, Benzoic acid; HCA, hydroxycinnamic acid. (**B**–**J**) Accumulation patterns of clusters I to IX. Clusters I to IX were specifically accumulated in maize (115 metabolites), adlay (127 metabolites), rice (152 metabolites), foxtail millet (130 metabolites), wheat (154 metabolites), common oat (105 metabolites), barley (124 metabolites), broomcorn millet (124 metabolites), and sorghum (100 metabolites). The x-axis depicts nine cereal crops, and the y-axis depicts the Z score standardized per metabolite.

To identify the patterns of metabolite abundance differences among the species, we performed hierarchical clustering analysis on the abundance of the 1131 metabolites across the nine crops, and a total of the metabolites were divided into nine clusters (Figure 5A). Analysis revealed that the metabolites enriched in these nine clusters were predominantly those with higher abundances in their respective crops compared to other crops: including 115 metabolites in maize, 128 metabolites in adlay, 153 metabolites in rice, 131 metabolites in foxtail millet, 154 metabolites in wheat, 105 metabolites in common oat, 124 metabolites in barley, 124 metabolites in broomcorn millet, and 101 metabolites in sorghum, respectively (Figure 5B–J). However, some metabolites, although enriched in specific clusters (corresponding to certain crops), exhibited higher abundances in other crops, such as 4 benzoxazinoids in cluster II (corresponding to adlay) showing higher levels in maize and to a lesser extent in adlay.

To identify the metabolic pathways enriched with the metabolites in each cluster, we performed enrichment analysis using the Kyoto Encyclopedia of Genes and Genomes (KEGG) for the metabolites in the nine clusters (Figure S1). Here, we focused on the enrichment of defense-related metabolic pathways in each cluster. Among the nine clusters, six were enriched in phenylpropanoid biosynthesis pathways, of which three were statistically significant (*p* < 0.05), and five were enriched in flavonoid biosynthesis pathways, of which three were statistically significant (*p* < 0.05). Additionally, benzoxazinoid biosynthesis pathway was enriched in cluster II (*p* = 0.098).

To better investigate metabolite abundance patterns in nine cereal crops, we compiled the classification of metabolites in each cluster (Table 1). Among the crops corresponding to the nine clusters, adlay (cluster II) was enriched in benzoxazinoids, phenolic acids, phytohormones, coumarins, and lignans. In rice (cluster III), phenolamines were found to be enriched. Foxtail Millet (cluster IV) was enriched in hydroxycinnamoyl derivatives, vitamins, phenolic acids, benzoic acids, and derivatives. Wheat (cluster V) was enriched in quinate and derivatives, flavonoids, and benzoxazinoids. The common oat (cluster VI) was enriched in lipids. Barley (cluster V) was enriched in benzoic acids and derivatives, coumarins and lignans, organic acids and derivatives, and alcohols. Broomcorn millet (cluster VII) was enriched in phenolamides, benzoic acids, and derivatives. Maize accumulated metabolites across multiple classes without a specific enrichment, and sorghum exhibited similar results.

### 2.4. Defense Metabolites Analysis

To better understand the reasons behind the differences in defense metabolites among the nine cereal crops, we integrated established pathways and proposed pathways to reconstruct detailed pathway maps of defense metabolites. These maps, combined with our metabolomic results, expanded our understanding of the current metabolic pathways in these crops.

**Figure 6:**
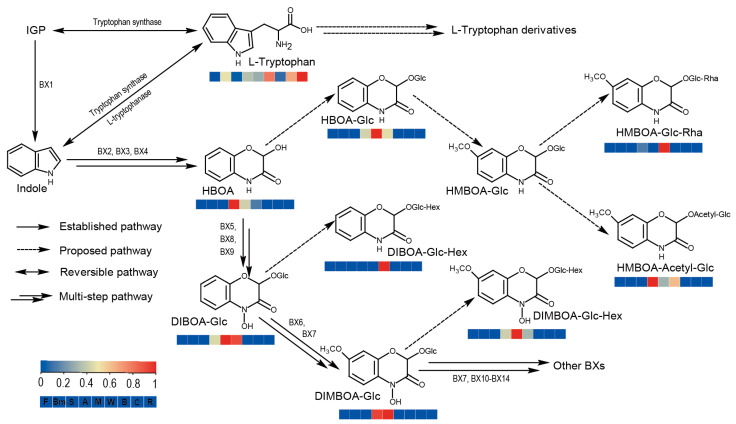
Metabolic pathway analysis for benzoxazinoid biosynthesis. BX1-14, benzoxazineless1 to benzoxazineless14, reported benzoxazinoid synthesis enzymes; Hex, hexose; Glc, glucoside; Rha, rhamnose.

We detected eight benzoxazinoids in maize, wheat, and adlay, among which the synthesis pathways for four compounds—HMBOA-acetyl-glucoside, HMBOA—glucoside-rhamnose, DIBOA-glucoside-hexose, and DIMBOA-glucoside-hexose—had not been identified. Therefore, we reconstructed a pathway (including established and proposed pathways) that includes the eight benzoxazinoids we detected (Figure 6). Considering the difference of one acetyl group, we hypothesize that HMBOA-acetyl-glucoside may be synthesized from HMBOA-glucose through the catalysis of an acetyltransferase. Similarly, HMBOA-glucoside-rhamnose, DIBOA-glucoside-hexose, and DIMBOA-glucoside-hexose are likely formed from their corresponding mono-glycosylated substrates through a single glycosylation reaction. By analyzing the concentrations of metabolites within these pathways, we found that HBOA-glucoside, DIBOA-glucoside, DIMBOA-glucoside-hexose, and DIMBOA-glucoside have relatively higher abundances in maize compared to adlay and wheat. These four metabolites can be synthesized from HBOA through 1–5 reaction steps (Figure 6). Wheat exhibited higher levels of DIBOA-glucoside-hexose and HMBOA-glucoside-rhamnose, while adlay showed higher levels of HBOA and HMBOA-acetyl-glucoside. In conclusion, based on our metabolomic results, we proposed previously unreported steps in the benzoxazinoid biosynthetic pathway and revealed both the similarities and differences of this pathway among maize, adlay, and wheat.

**Figure 7:**
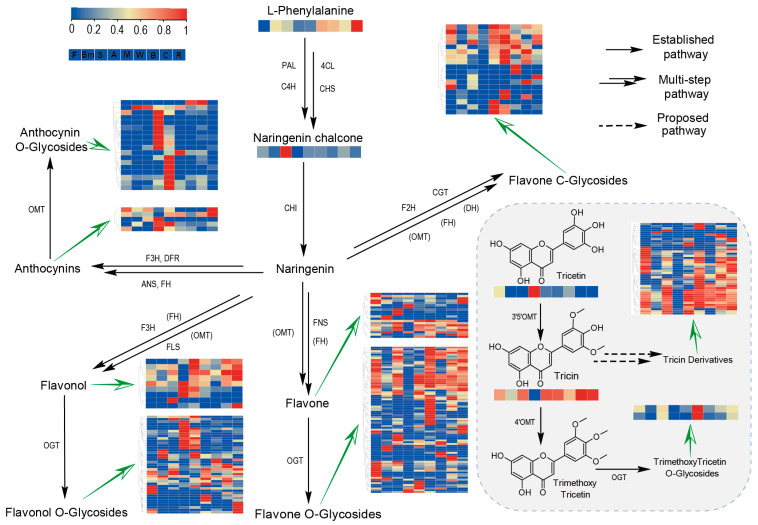
Metabolic pathway analysis for flavonoid biosynthesis. PAL, phenylalanine ammonialyase; 4CL, 4-coumarate-CoA ligase; C4H, cinnamic acid 4-hydroxylase; CHS, chalcone synthase; CHI, chalcone isomerase; F3H, flavonoid 3-hydroxylase; FH, flavonoid hydroxylase; F2H, flavonoid 2-hydroxylase; DH, dehydratase; DFR, dihydroflavonol 4-reductase; ANS, anthocyanin synthase; FLS, flavonol synthase; FNS, flavone synthase; OMT, O-methyltransferase; 3′5′OMT, 3′5′O-methyltransferase; 4′OMT, 4′O-methyltransferase; OGT, O-glycosyltransferase; CGT, C-glycosyltransferase. Parentheses indicate that only a part of the products requires this enzyme.

Additionally, we mapped the abundance of all the flavonoids we detected to the flavonoid biosynthesis pathway (Figure 7). Analysis revealed that adlay and maize contain more anthocyanins. Similarly, flavonol compounds with hydroxylation at the 3-position were enriched in adlay and maize, although this enrichment is not very apparent. Furthermore, flavonoid C-glycosides accumulated more in wheat and maize, whereas adlay showed the least accumulation. Analysis of all flavonoid O-glycosides indicated that adlay had the least accumulation, followed by maize, while no significant enrichment was observed in other cereal crops. Notably, unlike most flavonoids, tricetin—a highly hydroxylated flavonoid—was significantly enriched in adlay. Further analysis of tricetin-related compounds revealed that tricin (tricetin 3′,5′-dimethyl ether) and its derivatives were present at lower levels in adlay but accumulated more in wheat. Additionally, two trimethoxytricetin (TMT, 4′-methylated tricin) glycosides also accumulated heavily in wheat. In summary, we highlighted the differences in metabolites involved in each step of the flavonoid biosynthesis pathway among the nine cereal crops.

## 3. Discussion

Defense metabolites play crucial roles in protecting cereal crops from both biotic and abiotic stresses, thereby maintaining normal growth and yield. Throughout evolution, plants have developed diverse metabolic pathways, with specific metabolites playing significant roles in stress response [34,35,36]. In this study, comparative metabolomic analysis of nine cereal crops has revealed differences in the composition and abundance of defense-related metabolites, providing valuable insights into the variations in metabolism-mediated defense responses among different cereals.

As important defense metabolites, flavonoids are universally present in all plants [45]. With advancements in metabolome profiling methods, an increasing number of flavonoid derivatives have been discovered, and the functions of flavonoid compounds in plant defense are receiving heightened attention [18]. Previous studies have suggested that different subclasses of flavonoids play distinct roles in plants. In this study, we identified 311 flavonoids, and subsequent analyses revealed that flavonoids exhibit considerable diversity in both composition and abundance among cereal crops. The distinct metabolic profiles of different plant species may influence their adaptability to environmental stresses [46]. Thus, we hypothesize that flavonoids may be one of the primary contributors to the differences in environmental adaptability among cereal crops. Furthermore, flavone C-glycosides, a class of important phytoalexins, have been identified in various cereal crops, including rice, maize, wheat, sorghum, and barley [14,47,48]. Our study further explores the distribution of flavone C-glycosides in minor cereal crops, revealing that adlay millet has lower flavone C-glycoside content compared to other cereal crops. The distribution of tricetin and its derivative tricin in adlay, as well as the substantial accumulation of trimethoxytricetin in wheat, serve as exemplary cases. Notably, Zhu et al. [49] discovered a specific flavonoid 4′O-methyltransferase in wheat, enabling the synthesis of trimethoxytricetin, which is consistent with our findings. Similarly, we speculate that adlay accumulates tricetin but only a small amount of tricin, or even undetectable levels of TMT, likely due to the inhibition of enzymes responsible for converting tricetin to tricin. A recent study suggested that the presence of fiber in adlay seeds limits the bioavailability of its active ingredients, whereas microbial fermentation can enhance the content of various components in adlay seeds, including flavonoids [50]. The lower accumulation of flavonoid derivatives in adlay may be attributed to similar reasons. Considering that only a few flavonoids are species-specific and these flavonoids do not belong to specific subsets, we hypothesize that the majority of flavonoid modification pathways are present across all nine cereal crops. Whether these pathways function consistently across different species requires additional evidence.

In contrast to flavonoids, some metabolites are widely recognized as species-specific, such as glucosinolates, steroidal glycoalkaloids (SGAs), and isoflavones [20,34,35]. In this study, we also identified benzoxazinoids that are specifically present in maize, wheat, and adlay. The presence of these compounds in maize, wheat, and adlay is supported by extensive research [36,51]. Previous studies have primarily focused on the presence or absence of benzoxazinoids in various plants, their biosynthesis, and their bioactivities [37,52,53]. Our study addresses the abundance differences of benzoxazinoids in cereal crops, thereby complementing previous knowledge gaps. Specifically, four benzoxazinoids were found at high concentrations in maize, while wheat and adlay each had two benzoxazinoids with the highest concentrations. We found that DIMBOA-glucoside accumulated to higher levels in maize and adlay millet, a compound that has been reported to enhance the resistance of wheat and maize to aphids [54]. The benzoxazinoid biosynthesis pathway has been elucidated in maize, whereas only partial enzymes within this pathway have been reported in sorghum and foxtail millet. In contrast, genomic studies in rice and barley suggest the absence of genes required for benzoxazinoid biosynthesis [55]. There are no available reports for cultivated common oats and broomcorn millet. In the current understanding of benzoxazinoid pathways, the synthesis enzymes for the four benzoxazinoids we detected have not been reported, and our speculations on unknown pathways and abundance information provide references for the future discovery of these unknown synthesis enzymes. Moreover, although the main part of the benzoxazinoid pathway has been fully elucidated in maize, the differences in benzoxazinoid pathways between other plants such as wheat and adlay compared to maize are still insufficiently understood. The higher abundance of downstream HBOA substances in maize suggests that, relative to adlay and wheat, maize has a stronger ability to convert HBOA into these four substances or that these four substances are difficult to convert into other downstream benzoxazinoids in maize. Benzoxazinoids, in addition to serving as a class of important defense metabolites in maize, also play roles in regulating flowering time, auxin metabolism, iron uptake, and aluminum tolerance [52]. Our metabolomic data provide resources for a better understanding of the differences in benzoxazinoid producers.

A single plant contains over 5000 metabolites, most of which are specifically present in particular tissues, developmental stages, or environmental conditions [17,18]. Researchers have made efforts to identify variety-specific metabolites [56], metabolites specific to the entire life cycle of plants [57,58,59], and metabolites specific to particular environmental conditions [60], which have enhanced the understanding of plant metabolism. In this study, we focused on leaves at specific developmental stages of specific varieties. In future studies, we will examine metabolites of cereal crops in different tissues, at various developmental stages, and under diverse environmental conditions, thereby further elucidating the diversity of defense metabolites in cereal crops.

In recent years, genetic engineering has been widely applied to crop improvement [61]. For instance, in cotton, the introduction of the Bt gene enables the plant to produce Bt proteins that are toxic to specific pests, thereby enhancing the insect resistance of cotton [62]. Defensive metabolites hold similar potential for utilization. These metabolites have been found to play crucial roles in enhancing plants’ resistance to pests and diseases [25,28], drought tolerance [23,24], and UV-B resistance [14,63], making them important resources for future crop improvement. Unlike cotton, transgenic breeding of cereal crops for food purposes requires further consideration of edibility, which precludes the use of some potentially toxic metabolites as breeding targets [61]. The presence of benzoxazinoids in certain cereal crops illustrates their edibility and potential as transgenic breeding targets for benzoxazinoid-deficient crops like rice to improve insect resistance. Although the specific biological functions of most defensive metabolites in cereal crops are not yet fully under-stood, they hold promise as resources to be elucidated and utilized in the future.

In summary, our comparative metabolomic analysis revealed differences in defense metabolites, including flavonoids and benzoxazinoids, among nine cereal crops. This not only provides new insights into the differences in metabolic pathways among various cereal crops but also offers metabolic resources that can be referenced for understanding the environmental adaptability and stress tolerance differences among cereal crops.

## 4. Materials and Methods

### 4.1. Plant Materials and Growth Conditions

To understand the metabolic characteristics of cereal crops, we selected nine major or minor cereal crops and additionally included soybean as an outgroup. The nine selected crops are rice, wheat, maize, barley, sorghum, common oats, foxtail millet, broomcorn millet, and adlay. All materials were cultivated in specific artificial climate chambers at the Sanya Institute of Breeding and Multiplication, Hainan University. Rice was grown using hydroponics with the standard nutrient solution described previously [64]. Other crops were cultivated in nutrient soil (a 2:1 mixture of soil and perlite). The growth conditions were as follows: for wheat, barley, and common oat, grown at 25 °C during the day and 22 °C at night; for other crops, grown at 28 °C during the day and 25 °C at night. All plants were subjected to a light cycle of 14 h of light and 10 h of darkness, with a humidity level of 65%. Watering or nutrient solution replacement was performed every two days. As described previously, when the crops reached the five-leaf stage, at least three mature leaves from individual plants were collected as one biological replicate for each crop, with three biological replicates per crop [65]. Mature leaves were collected at the five-leaf stage, with three biological replicates for each crop, and each biological replicate consisted of a mixed sample from at least three plants. Immediately after collection, all samples were flash-frozen in liquid nitrogen and then stored at −80 °C for future use.

### 4.2. Chemical Reagents

Chromatographic-grade acetonitrile and methanol required for mass spectrometry were purchased from Thermo Fisher (Waltham, MA, USA) (https://www.thermofisher.cn/, accessed on 11 October 2024). Ultrapure water was purified using a Millipore purification system (Millipore Corporation, Burlington, MA, USA). Acetic acid, lidocaine, and standard reference compounds required for identification were obtained from Macklin (Shanghai, China) (https://www.macklin.cn/, accessed on 25 July 2024) and Sigma (Setagaya, Japan) (http://www.sigmaaldrich.com/, accessed on 25 July 2024). All standard compounds were dissolved in DMSO to prepare stock solutions and then diluted with 70% methanol–water to appropriate concentrations for LC-MS analysis.

### 4.3. Sample Preparation and Extraction

Samples stored at −80 °C were subjected to freeze-drying using a lyophilizer (SCIENTZ-100F/A, Ningbo, China, https://www.scientz.net/, accessed on 20 September 2024) for seven days. The lyophilizer maintained a constant vacuum of 0 mTorr throughout the process, with the following temperature program: −30 °C for 10 h; −20 °C for 20 h; −5 °C for 60 h; 0 °C for 30 h; 10 °C for 20 h; and 26 °C for 28 h. The dried samples were then ground using a mixer mill (MM 400; Retsch, Haan, Germany) at a speed of 30 Hz for 1 min.

Metabolite extraction was performed according to our previously described method [42]. For each sample, 100 mg was weighed into a 2 mL centrifuge tube, and 1 mL of pre-cooled 70% aqueous methanol (containing 0.01 mg/L lidocaine as an internal standard) was added. The mixture was vortexed for 15 s and allowed to stand for 10 min, and this process was repeated three times before the samples were placed in a 4 °C refrigerator to stand overnight. The next day, the samples were centrifuged at 10,000× *g* for 10 min, filtered through a 0.22 μm filter (SCAA-104, ANPEL, Shanghai, China, http:// www.anpel.com.cn, accessed on 12 August 2024), and transferred to LC-MS vials for analysis. Additionally, a 10 μL aliquot from each sample was pooled to create a quality control sample to assess the stability of the instrument.

### 4.4. LC–MS/MS Analysis

Untargeted metabolomic analyses were performed using an LC-ESI-QqTOF-MS/MS (TripleTOF 6600+, Applied Biosystems, Waltham, MA, USA) operating in TOF MS/MS mode. The relevant parameters were: Gas1, 50 psi; Gas2, 60 psi; curtain gas, 35; interface temperature, 550 °C; positive ion spray voltage, 5500 V; negative ion spray voltage, 4500 V; scan range from *m*/*z* 80 to *m*/*z* 1000; accumulation time for MS1, 0.2 s; and total cycle time, 0.8 s. Information-Dependent Acquisition (IDA) settings were: collision energy (CE) of 35 V, collision energy spread (CES) of 15 V, triggering up to 15 MS/MS ions, and an MS/MS accumulation time of 0.5 s.

Targeted metabolomic analyses were conducted using an LC-ESI-QTRAP-MS/MS (QTRAP 6500+, Applied Biosystems, Waltham, MA, USA) operating in Multiple Reaction Monitoring (MRM) mode. The relevant parameters were interface temperature of 450 °C; cycle time of 0.8 s; and minimum dwell time of 3 milliseconds, with other mass spectrometry parameters consistent with those used in untargeted analyses.

Both untargeted and targeted metabolomic analyses utilized a CBM40A liquid phase system (Shimadzu, Kyoto, Japan) under the following conditions: chromatography column, Waters ACQUITY UPLC HSS T3 C18 column (2.1 mm × 100 mm, 1.8 μm); solvent system, water (0.04% acetic acid): acetonitrile (0.04% acetic acid); elution gradient, 95:5 *v*/*v* at 0 min, 5:95 *v*/*v* at 10.0 min, 5:95 *v*/*v* at 12.0 min, 95:5 *v*/*v* at 12.1 min, and 95:5 *v*/*v* at 15.0 min; flow rate, 0.35 mL/min; column temperature, 40 °C; injection volume, 5 μL for untargeted metabolomic analyses and 2 μL for targeted metabolomic analyses. During LC-MS analysis, the quality control sample was analyzed every 10 injections to evaluate the reproducibility of the quality control sample and the internal standard, thereby assessing the reliability of the data.

### 4.5. Metabolome Data Processing

For untargeted metabolomics data, signal extraction and peak alignment were performed using MS-DIAL (version 5.2.4) [40]. Signals with a sample-to-blank response ratio > 5 were retained after background subtraction, and signals with peak height > 500 and a signal-to-noise ratio > 10 were filtered. Based on the filtered signals, MS^2^ data were screened using Python (version 3.10) to form an MS^2^ spectral tag library (MS2T). Metabolite identification was then performed based on accurate *m*/*z* values, retention times (RT), and MS^2^ spectra. For targeted metabolomic data, quantitative analysis was conducted using MultiQuant 3.0.3 software (Applied Biosystems, Waltham, MA, USA). Signals with a signal-to-noise ratio > 10 were filtered, and peak areas were exported for subsequent analysis.

### 4.6. Statistical Analysis

Metabolomics data were assessed for reproducibility using quality control samples. Features that passed the evaluation (coefficient of variation ≤ 30%) were averaged across three biological replicates and used for subsequent analysis. Principal component analysis (PCA) was performed on log2-transformed data to enhance data uniformity, and hierarchical clustering was carried out on Z-score transformed data. PCA and hierarchical clustering analyses were conducted using OmicStudio tools (http://www.omicstudio.cn/tool, accessed on 21 December 2024) [66]. For the distribution of major secondary metabolite classes, the detection proportion of each metabolite class in each crop was calculated as the ratio of detected metabolites of that class in the crop to the total number of metabolites in that class. The coefficient of variation for each metabolite class was calculated based on the detection proportions of that class across the nine crops.

### 4.7. Identification and Analysis of Metabolic Pathway from Cluster I to Cluster IX

The metabolites in clusters I to IX were annotated by searching the KEGG database (http://www.kegg.jp/kegg/pathway.html, accessed on 20 December 2024) for their corresponding KEGG IDs. KEGG enrichment analysis was performed using MBROLE 2.0 (http://csbg.cnb.csic.es/mbrole2/, accessed on 24 December 2024), with statistical significance determined by Student’s *t*-test (*p* ≤ 0.05 considered significant), followed by visualization in OmicStudio (accessed on 24 December 2024).

## 5. Conclusions

This study reveals the metabolic diversity of defense metabolites in nine cereal crops, identifying 1131 annotated metabolites across 18 classes. We found 531 common metabolites shared among all species, with each crop possessing unique metabolites. Notably, eight benzoxazinoids were uniquely present in maize, wheat, and adlay. Pathway analysis highlighted the enrichment of stress-related flavonoid metabolism, with maize showing a high accumulation of HBOA derivatives. These findings provide valuable insights into the metabolic profiles of cereals, contributing to the understanding of stress resistance mechanisms and aiding efforts to improve cereal crop resilience.

## Figures and Tables

**Table 1:** Distribution of the metabolites among nine clusters.

	Cluster I	Cluster II	Cluster III	Cluster IV	Cluster V	Cluster VI	Cluster VII	Cluster VIII	Cluster IX	In Total
	Maize	Adlay	Rice	Foxtail Millet	Wheat	Common Oat	Barley	Broocorn Millet	Sorghum	
AA and derivatives	7	7	36	8	10	4	14	7	8	101
Alcohols and polyols	3	9	2	6	6	5	2	10 *	5	48
Alkaloids	9	4	15	6	6	4	10	4	6	64
BA and derivatives	0	2	0	3 *	2	2	3 *	1	1	14
Benzoxazinoids	0	6 **	0	0	2 *	0	0	0	0	8
Coumarins and lignans	3	8 *	1	5	4	0	2	9 *	7	39
Flavonoids	35	36	32	34	69 *	24	22	23	36	311
HCA derivatives	3	8	7	15	4	2	8	9	3	59
Lipids	31	3	9	14	16	43 *	30	20	6	172
Nucleotides and derivates	8	4	9	3	6	3	5	3	7	48
Organic acids	2	10	6	9	3	6	3	12 *	6	57
Others	4	5	4	2	6 *	1	4	3	1	30
Phenolamides	0	7	15 *	1	2	1	9	11 *	0	46
Phenolic acids	3	7 *	1	7 *	5	1	1	4	4	33
Phytohormones	4	6 *	2	4	3	2	1	2	3	27
Quinate and derivatives	2	1	2	1	4 *	2	0	2	2	16
Terpenoids	0	2	5	5	2	3	6 *	2	1	26
Vitamins	1	2	6	7 *	4	2	4	2	4	32
In total	115	127	152	130	154	105	124	124	100	1131

AA, amino acid; BA, Benzoic acid; HCA, hydroxycinnamic acid. * Accounted for more than 20%. ** Accounted for more than 40%.

## Data Availability

Data supporting the findings of this study are available within the manuscript and Supplemental Information Files or are available from the corresponding author upon request.

## Supplementary Materials

The following supporting information can be downloaded at: https://www.mdpi.com/article/10.3390/plants14040629/s1. Figure S1: KEGG (Kyoto Encyclopedia of Genes and Genomes) analysis of clusters I to IX based on metabolites. (A-I) KEGG analysis of clusters I to IX, which correspond to metabolites accumulated in maize, adlay, rice, foxtail millet, wheat, common oat, barley, broomcorn millet, and sorghum. Each cluster displays only the top 20 pathways with the smallest *p*-values. Table S1: Information of 1131 Annotated Metabolites in This Study. Table S2: Statistics of Shared Metabolites Among Nine Cereal Crops. Table S3: List of Species-Specific Metabolites for Each Cereal Crop. Table S4: List of Metabolites Missing in Only One Species. Table S5: Number and Proportion of Detected Metabolites in Major Classes Across Various Cereal Crops.
