# Supplementary material for: Metabolomic Analysis Reveals the Diversity of Defense Metabolites in Nine Cereal Crops
Source: Plants (Basel). 2025 Feb 19;14(4):629. doi: 10.3390/plants14040629 (PMC11859589; doi:10.3390/plants14040629)
Supplement: Supplementary file 1 [file plants-14-00629-s001.zip › Supplemental information_Proofread/Supplemental Figure with captions.docx]

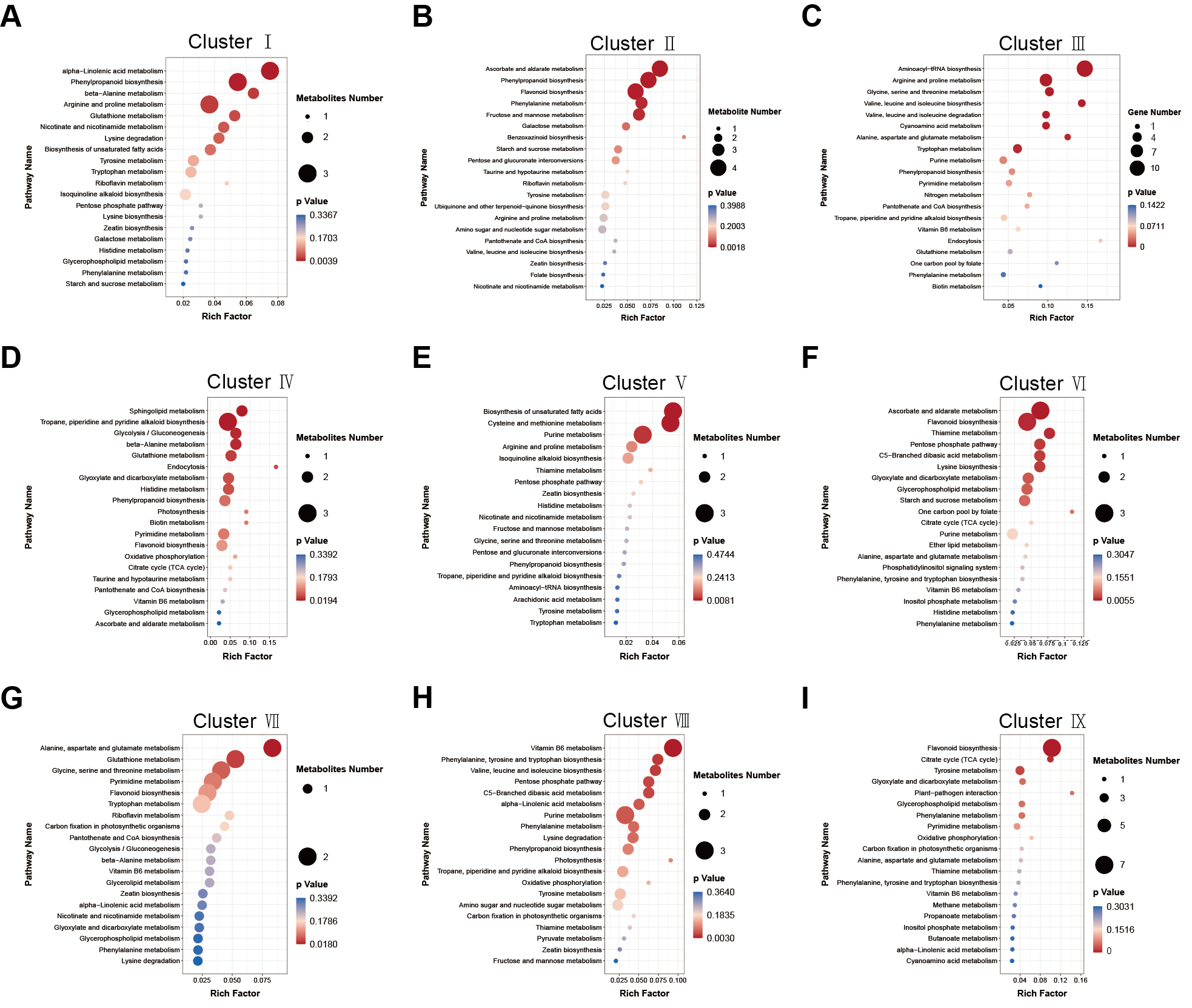


**Figure S1.** **KEGG (Kyoto Encyclopedia of Genes and Genomes)** **analysis of clusters I to IX based on metabolites.** (A-I) KEGG analysis of clusters I to IX, which correspond to metabolites accumulated in maize, adlay, rice, foxtail millet, wheat, common oat, barley, broomcorn millet, and sorghum. Each cluster displays only the top 20 pathways with the smallest p-values.
